# Supplementary material for: Utility of multimodal longitudinal imaging data for dynamic prediction of cardiovascular and renal disease: the CARDIA study
Source: Front Radiol. 2024 Feb 27;4:1269023. doi: 10.3389/fradi.2024.1269023 (PMC10927728; doi:10.3389/fradi.2024.1269023)
Supplement: Supplementary file 1 [file Datasheet1.pdf]

Supplementary Material

Table S1. Markers used for prediction in this study
Table S2. Different imaging variable subset combinations, ranked by the averaged iAUC gain with respect to the baseline model that was trained on using traditional risk factors

Figure S1. Cumulative incidence function of the outcome (Cardiovascular and Renal Disease)

Figure S2. Comparison of modeling methods

Table S1. Markers used for prediction in this study

Modality	Exam Year	Variable
**Echo**	Y5, Y10 (substudy), Y20, Y25	LA (Area, Dimension, Volume); LV (Internal Dim, Volume, EF, Post Wall Thickness); Stroke Volume; Aortic Root Dimension; Ventricular Septal Thickness; Peak Strain (Radical, Circumferential Longitudinal); Global LV Hypertrophy; Valve Condition (Regurgitation, Stenosis, Prolapse) (for Aortic, Mitral, Tricuspid); Mitral Valve E-wave To A-wave Ratio; Heart Rate; Doppler E-wave Prime Velocity; RV (S-wave Velocity, Systolic Pressure); Tricuspid Annular Peak Systolic Excursion
**CT**	Y10 (substudy), Y15, Y20, Y25	Mean/Mass/Volume/No. of Lesions and Agatston Score of {Whole Heart, LAD, Left Circumflex, Left Main, Right Coronary, All Coronary}; Coronary Artery Calcium (CAC) Scoring (Y/N, Total Agatston Score); Presence of Pacemaker, Stent, Valve Replacement, Previous Cardiac Surgery, Coronary Artery Bypass Graft Pericardial Fat (Mean Attenuation, Standard Deviation, Volume of Pericardial Adipose Tissue); Abdominal Fat (Volume of Total Abdomen Adipose Tissue (TAAT), Visceral Adipose Tissue (VAT), Intermuscular Adipose Tissue (IMAT), Subcutaneous Adipose Tissue (Sat), Total Abdomen); Hepatic Steatosis (Mean Attenuation, Standard Deviation, Volume Of Liver Fat); Mean/Calcium Mass/Volume/No. of Lesions and Agatston Score of {Left Common Iliac, Right Common Iliac, Infrarenal Abdominal Aorta}; Aorto-iliac Calcium Scoring (Y/N, Agatston Score)
**DEXA**	Y7 (ancillary), Y20	Bone Mass Density (BMD) and % Fat of {Whole Body, Head, Trunk, Arms, and Legs}
**Carotid IMT**	Y25	Average maximum and mean Intima-media Thickness of {common carotid artery, carotid artery bulb, and internal carotid artery}
**Brain MRI**	Y25 (substudy), Y30 (substudy)	Abnormal Tissue Volume/Corrected Mean Of Fa Values/Total Volume/Mean Of Cerebral Blood Flow/Mean % Change Of Vascular Reactivity Of {White Matter, Gray Matter, Cerebrospinal Fluid, Superior Sagittal Sinus, and Total Brain}
**Traditional risk factors**	All Years	9 ASCVD variables (Age, Sex, Race, SBP, CHOL, HDL, Hypertensive Med, DM, Smoking Status) and 6 additional risk factors (DBP, BMI, CHOL-lowering med, LDL, Triglycerides, Fasting glucose)

Echocardiography (Echo), Carotid Intima-Media Thickness (CARTD), Dual-Energy X-ray Absorptiometry (DEXA), and Brain Magnetic Resonance Imaging (MRI).

Table S2. Different imaging variable subset combinations, ranked by the averaged iAUC gain with respect to the baseline model that was trained on using traditional risk factors

**Feature subset**	**Averaged iAUC gain**
Traditional risk factors and all_imaging	0.0291
Traditional risk factors and echo_ct_mri	0.0274
Traditional risk factors and echo_ct_dexa_cartd	0.0257
Traditional risk factors and echo_ct_dexa_mri	0.0246
Traditional risk factors and echo_ct_cartd_mri	0.0232
Traditional risk factors and echo_ct_dexa	0.0232
Traditional risk factors and echo_ct_cartd	0.0230
Traditional risk factors and echo_ct	0.0218
Traditional risk factors and echo_dexa_mri	0.0215
Traditional risk factors and ct_dexa	0.0196
Traditional risk factors and ct_cartd_mri	0.0187
Traditional risk factors and echo_mri	0.0182
Traditional risk factors and echo_cartd_mri	0.0171
Traditional risk factors and ct_dexa_cartd_mri	0.0168
Traditional risk factors and echo_dexa_cartd	0.0164
Traditional risk factors and echo_cartd	0.0152
Traditional risk factors and ct_dexa_cartd	0.0150
Traditional risk factors and ct_mri	0.0144
Traditional risk factors and ct_dexa_mri	0.0142
Traditional risk factors and ct	0.0137
Traditional risk factors and echo_dexa_cartd_mri	0.0137
Traditional risk factors and ct_cartd	0.0135
Traditional risk factors and echo	0.0108
Traditional risk factors and dexa_cartd_mri	0.0088
Traditional risk factors and cartd_mri	0.0083
Traditional risk factors and dexa	0.0082
Traditional risk factors and echo_dexa	0.0067
Traditional risk factors and mri	0.0061
Traditional risk factors and dexa_mri	0.0022
Traditional risk factors and dexa_cartd	-0.0001
Traditional risk factors and cartd	-0.0020

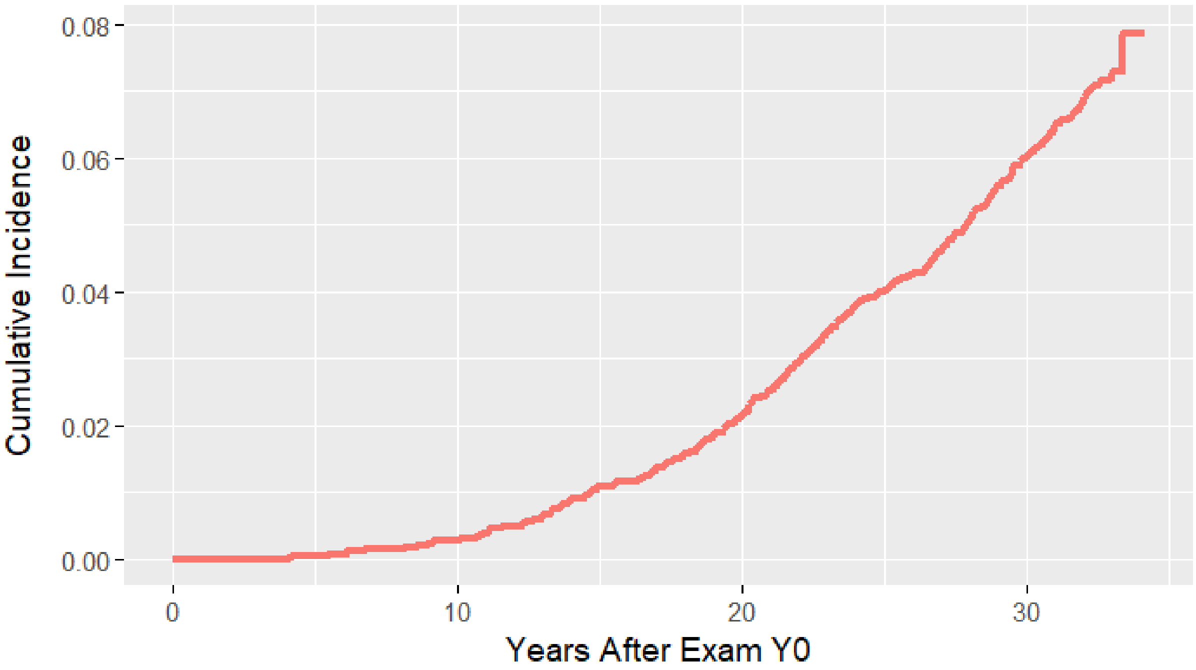

Figure S1. Cumulative incidence function of the outcome (Cardiovascular and Renal Disease)

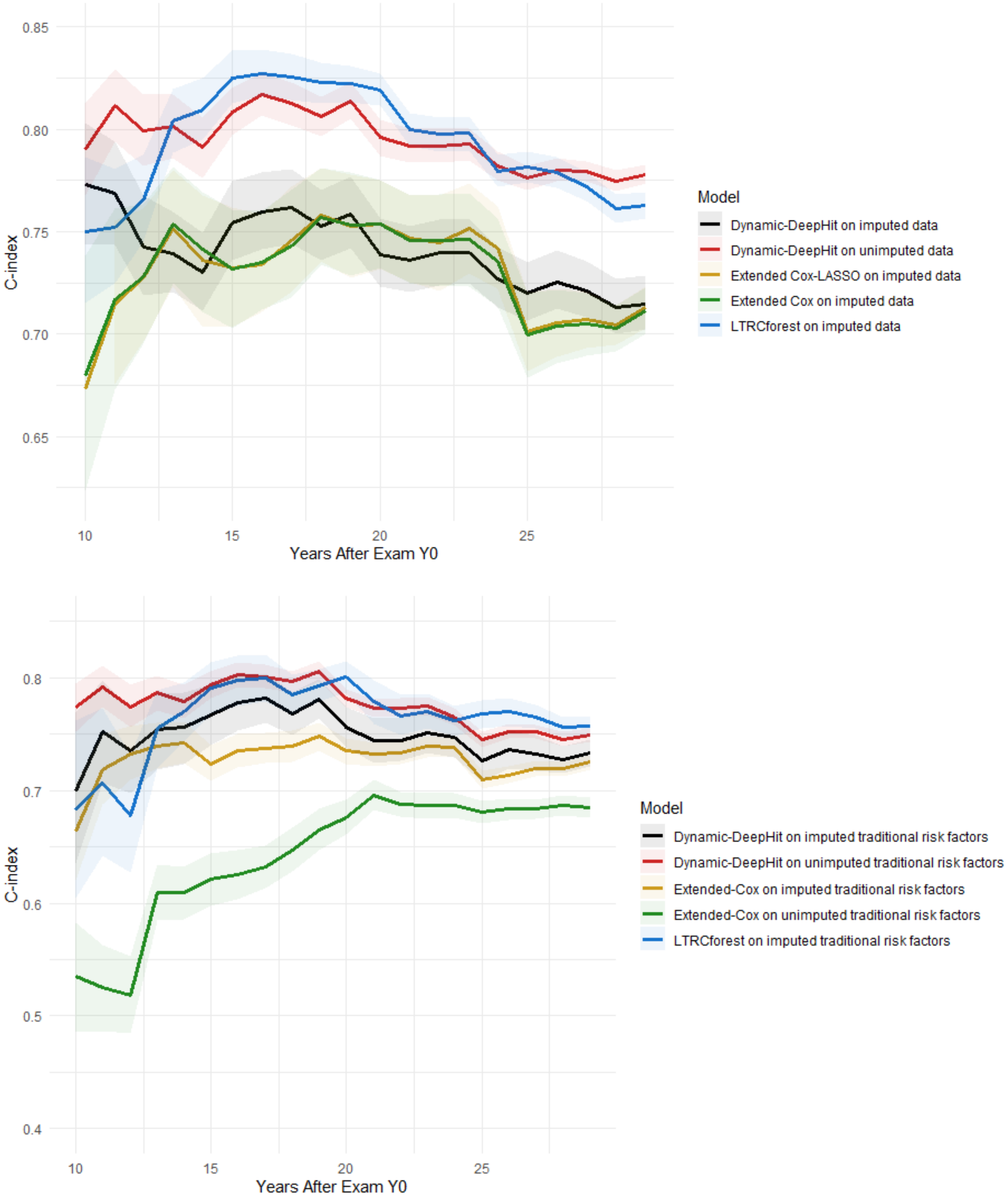

Figure S2. Comparison of modeling methods. Top: trained on all variables. Bottom: trained on traditional risk factors. When training on all variables, 3 models failed to converge (LTRCforest, Extended Cox, and Extended Cox-LASSO on unimputed data) because of high missing rates. Dynamic-DeepHit on unimputed data and LTRCforest on imputed data are consistently the best, whereas Extended Cox consistently underperformed. Dynamic-DeepHit on unimputed data and LTRCforest on imputed data are the best.
